# Poly(Propylene Carbonate)-Based Biodegradable and Environment-Friendly Materials for Biomedical Applications

**DOI:** 10.3390/ijms25052938

**Published:** 2024-03-02

**Authors:** Li Wang, Yumin Li, Jingde Yang, Qianqian Wu, Song Liang, Zhenning Liu

**Affiliations:** Key Laboratory of Bionic Engineering (Ministry of Education), College of Biological and Agricultural Engineering, Jilin University, Changchun 130022, China

**Keywords:** poly(propylene carbonate) or PPC, biomaterials, modification, biomedical application, drug carriers, wound dressings, implants

## Abstract

Poly(propylene carbonate) (PPC) is an emerging “carbon fixation” polymer that holds the potential to become a “biomaterial of choice” in healthcare owing to its good biocompatibility, tunable biodegradability and safe degradation products. However, the commercialization and wide application of PPC as a biomedical material are still hindered by its narrow processing temperature range, poor mechanical properties and hydrophobic nature. Over recent decades, several physical, chemical and biological modifications of PPC have been achieved by introducing biocompatible polymers, inorganic ions or small molecules, which can endow PPC with better cytocompatibility and desirable biodegradability, and thus enable various applications. Indeed, a variety of PPC-based degradable materials have been used in medical applications including medical masks, surgical gowns, drug carriers, wound dressings, implants and scaffolds. In this review, the molecular structure, catalysts for synthesis, properties and modifications of PPC are discussed. Recent biomedical applications of PPC-based biomaterials are highlighted and summarized.

## 1. Introduction

Polymers are now widely used as biomaterials in clinics and scientific research. Among them, synthetic polymers have become a major source of biomedical materials due to their outstanding mechanical properties, structural maneuverability and processability. They can be tailored and modified to meet the demand of various applications, such as artificial skin, implanted scaffolds and drug delivery systems and are thus regarded as very promising alternative biomaterials in healthcare [1,2].

Poly(propylene carbonate) (PPC) is an aliphatic polycarbonate with the advantages of low-cost, low-toxicity, environmental friendliness and biodegradability. PPC was first synthesized by Inous in a ZnEt_2_/H_2_O-catalyzed system through the copolymerization of CO_2_ and propylene oxide (PO) [3]. The immobilization of CO_2_ as a feedstock into PPC will not only reduce the usage of petrochemicals, but also mitigate the environmental problems caused by greenhouse gases. Such a “carbon fixation” function makes PPC an ideal polymer for the era of “carbon neutrality”.

Hence, various homogeneous and heterogeneous catalysts have been developed over recent decades to synthesize PPC with enhanced properties and productivity to achieve broader applications [4,5,6]. At present, PPC has been extensively used in food packaging [7,8], battery manufacturing [9,10], agricultural mulch films [11] and cushioning foams [12], etc. In addition, owing to its good biocompatibility and non-toxic degradation products, PPC also holds significant promise for biomedical applications, such as drug carriers [13,14], tissue engineering scaffolds [15,16] and medical dressings [17].

However, the use of PPC in medicine is still hindered by some drawbacks, such as its hydrophobic nature, fast degradation, poor mechanical strength, low thermal stability and glass transition temperature. Thus, various approaches have been taken to modify PPC in order to overcome these disadvantages. In this review, we will provide a brief overview of the use of biomaterials in healthcare focusing on biodegradable synthetic polymers, and then summarize the synthesis, properties, modification and applications of PPC. In particular, the recent advances in the biomedical applications of PPC-based biomaterials are highlighted, including drug carriers, wound dressings and implants.

## 2. Biomaterials Based on Synthetic Polymers

Broadly speaking, biomaterials are any type of medical material that can improve and enhance human life and health. Since the 1940s, biomaterials have made evident developments with the advances in regenerative medicine and materials science [18,19]. Biomaterials for the applications of artificial skin, cardiac and vascular scaffolds, neural scaffolds, prosthetic replacements, drug delivery vehicles and others have evolved from basic biocompatible claims to current immunomodulatory treatments. It should be noted that while biocompatibility is the most fundamental attribute for biological materials, the chemical composition, mechanical properties, degradation properties, antibacterial activities and material–host interactions should also be a focus of concern.

### 2.1. Synthetic Polymers as Biomaterials

Synthetic polymers have attracted the interest of researchers in various fields due to their technical flexibility, maneuverable chemical structure, commercial accessibility and desirable mechanical properties. Generally speaking, synthetic polymers can be divided into biodegradable and inert (undegradable) categories according to the degradation rate and pathway. From a clinical perspective, biodegradable materials are more attractive than undegradable ones because their advantages of bio-absorbability can avoid the need for a second surgery. Biodegradable polymers usually contain chemical bonds that are susceptible to hydrolysis or degradation by enzymes in living organisms in their main chains [20,21]. Common bonds include esters, amides and carbonates. Among them, aliphatic polyesters, whose chemical structure and composition can be easily modified to achieve hydrophilicity, pro-cell adhesion, proliferation and growth, pro-neovascularization, pro-tissue regeneration and drug targeting, have been regarded as “a promising alternative” in tissue engineering [1,22,23].

**Figure 1:**
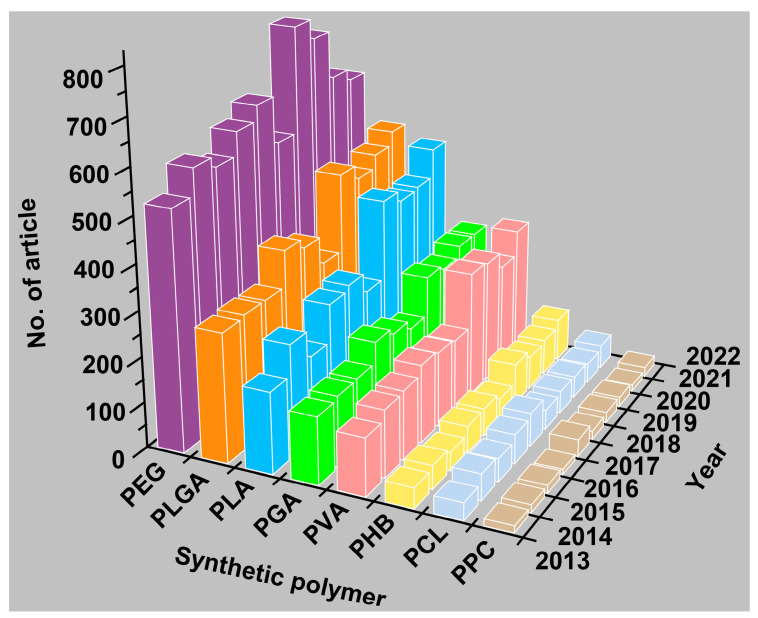
The numbers of publications on PEG, PLGA, PLA, PGA, PVA, PHB, PCL or PPC in healthcare over the last decade.

Over the recent decades, biodegradable synthetic polymers have been extensively used as biomaterials, including disposable medical devices, implantable stents, skin substitutes, targeted drug carriers, gene and siRNA delivery vehicle, and so on. For example, polylactic acid (PLA), a hydrophobic and thermally stable aliphatic polyester that biodegrades into non-toxic natural lactic acid, has been widely used in biomedical applications. PLA can be used alone or in combination with other small molecules or polymers for cartilage and joint regeneration [24,25], drug delivery and release [26,27], wound healing [28,29] and other therapies [30,31,32,33,34,35,36]. Other biodegradable polymers, including poly(lactide-co-glycolic acid) (PLGA), poly(vinyl alcohol) (PVA), poly(ε-caprolactone) (PCL), poly(glycolic acid) (PGA), poly(3-hydroxybutyrate) (PHB), poly(ethylene glycol) (PEG) and PPC (Figure 1) [37,38,39], have also been applied in healthcare.

### 2.2. Preparation of Synthetic Polymers as Biomaterials

Several manufacturing techniques, such as electrospinning, electrospraying and electrophoretic deposition, have been used to prepare biomaterials from synthetic polymers. For instance, electrospinning has been adopted to fabricate nanofilms or composite films from PEG [40], PLGA [41], PLA [42], PGA [43], PVA [44], PHB [45], PCL [46] and PPC [47] for the applications of wound dressings and drug delivery systems. Deng et al. prepared core-shell nanofibers of “spindles-on-a-string (SOS)” by coaxial electrospinning. Both the core of ethyl cellulose and the shell of PEG contained ibuprofen, which ensures a well-controlled release of the drug [40].

More recently, 3D printing has been used to build scaffolds for the repair and/or regeneration of various tissues, including bone, cartilage, nerve and blood vessels [48], owing to its advantages of flexibility in topology design and precision in manufacturing. Many synthetic polymers have been 3D-printed, such as PEG [49], PLGA [50], PLA [25] and their composites. For example, Zhang et al. prepared composite scaffolds of PLA and nano-hydroxyapatite (n-HA) via 3D printing. Compared to pure PLA scaffold, the PLA/n-HA scaffold demonstrated better mechanical properties, biocompatibility and osteogenesis, which is promising for the repair of large bone defects [25].

In addition, some researchers have combined several manufacturing technologies to create polymeric materials with different dimensions, shapes, sizes, structures and functions. The combination approach leverages the advantages of each manufacturing technology and thus holds significant potential for various biomedical applications. For example, Zhou et al. dispersed electrospun gelatin-PLGA fibers in a bioink derived from a decellularized cartilage matrix to construct a scaffold by 3D printing. The scaffold not only exhibited excellent mechanical properties, but also enhanced cell infiltration, cell growth and cartilage regeneration [51].

## 3. Synthesis and Chemical Structure of PPC

The use of degradable plastics is becoming increasingly important for environmental protection. PPC is an aliphatic biodegradable polymer with a “carbon fixation” function and thus has great development potential in the era of “carbon neutrality”.

### 3.1. Synthesis of PPC

In recent years, CO_2_-based polymers, represented by PPC, have gained increasing recognition and attention. Since CO_2_ is the major greenhouse gas, using CO_2_ as a raw material can not only mitigate the greenhouse effect caused by the consumption of fossil fuels, but also effectively utilize renewable carbon resources. Additionally, CO_2_-based polymers are usually degradable. So, from these perspectives, the chemical fixation of CO_2_ in the synthesis of PPC is in line with the concept of sustainable development and “green chemistry”.

**Figure 2:**
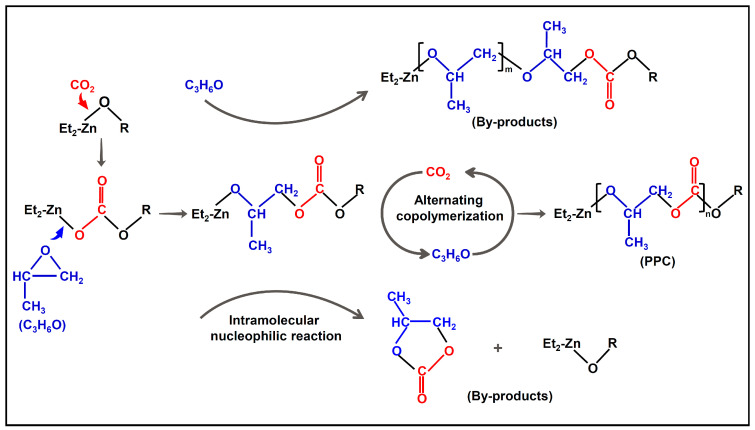
Reaction scheme for the synthesis of PPC via alternating copolymerization of propylene oxide (C_3_H_6_O, PO) and CO_2_. (Red and blue represent CO_2_ and PO motifs, respectively.).

PPC was originally synthesized by Inous in a ZnEt_2_/H_2_O-catalyzed system through the copolymerization of CO_2_ and PO [3], as shown in Figure 2. Briefly, the reaction between ZnEt_2_ and H_2_O first forms an intermediate with a -Zn-O-R motif, and then the elongation of the PPC chain is achieved by alternating insertion of CO_2_ and PO at the Zn-O bond.

**Figure 3:**
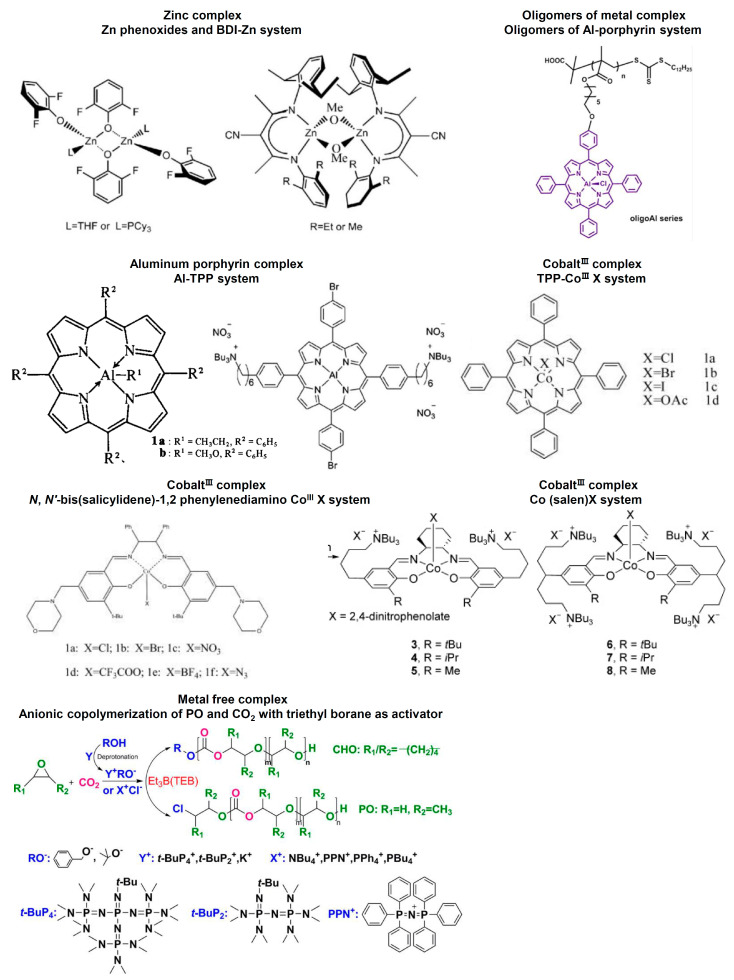
Representative homogeneous catalysts, such as zinc complex [4,53,54,55], aluminum porphyrin complex [56,57], cobalt^III^ complex [58,59,60], oligomers of metal complex [62,63], and metal free complex [64] for the synthesis of PPC.

A series of catalysts have been developed to extend the chain length of PPC in order to enhance the properties of PPC as well as to reduce the cost of synthesis. Currently, both homogeneous and heterogeneous catalysts have been developed to improve the efficiency of PPC synthesis (Table 1). Compared to heterogeneous catalysts, homogeneous catalysts are more favored by researchers for their clearer chemical structure, higher activity and tunability [4,52]. Common homogeneous catalysts for PPC synthesis include the complexes of zinc (Zn), aluminum (Al) or cobalt^III^ (Co^III^) (Figure 3). The complexes of Zn are relatively old catalysts with a low polymerization rate or selectivity [53,54,55]. Al-based complexes, such as Al-tetraphenylporphyrin (Al-TPP), used to have low activity [56]. However, Wang and co-workers recently modified Al-TPP with substitutes at the para-position of the phenyl rings to improve the activity [57]. Over the recent decades, the Co^III^-based complex has become a rising star for the catalysis of PPC synthesis. Co^III^ TPP chloride (TPPCo^III^Cl) and *N*, *N*′-bis(salicylidene)-1,2 phenylenediamino Co^III^ X (X = ${Cl}^{-}$, ${Br}^{-}$, ${NO}_{3}^{-}$, $N_{3}^{-}$, ${CF}_{3}{COO}^{-}$ and ${BF}_{4}^{-}$) have shown high turnover frequency (TOF) and selectivity in PPC synthesis [58,59]. A more recent work with Co^III^(salcy)X has demonstrated even better performance [60]. It should be noted that the complexes of other metals, such as titanium^IV^ and chromium^III^, have also exhibited good catalytic activity in a similar reaction between cyclohexene oxide and CO_2_ [61].

In recent years, oligomeric metal complexes have been developed to achieve intramolecular multisite cooperativity and to improve the stability and activity of homogeneous catalysts (Figure 3). For example, Wang and co-workers designed several oligomeric catalysts anchored with Al-porphyrin complexes through reversible addition-fragmentation chain transfer polymerization, which demonstrated superior activities [62,63]. Another interesting trend is to use metal-free catalysts in PPC synthesis, such as onium halides or onium alkoxides (Figure 3) [64]. However, the activities of metal-free catalysts are still relatively low.

**Figure 4:**
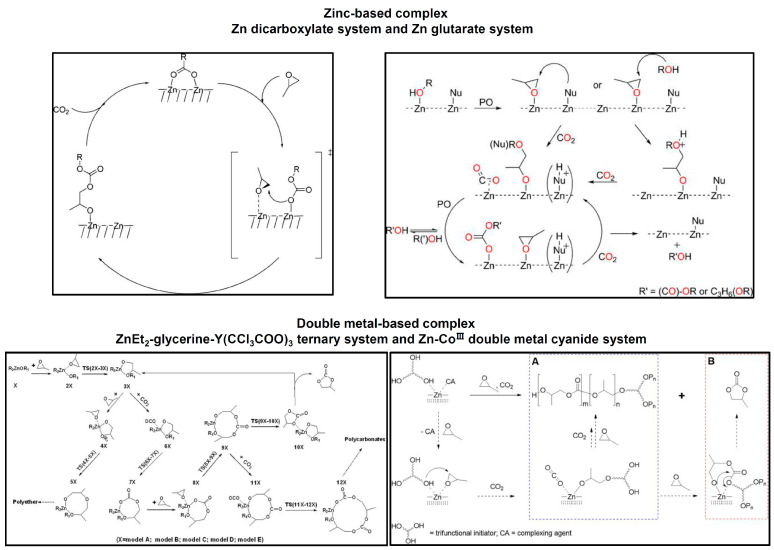
Representative heterogeneous catalysts, such as zinc-based complex [65,66], and double metal-based complex [68,69] for the synthesis of PPC. (A represents route for the synthesis of PPC. B represents route for the production of byproduct, predominantly cyclic propylene carbonate.).

Heterogeneous catalysts are less used in PPC synthesis due to the fact that their chemical structures or underlying copolymerization mechanisms have not been completely elucidated. The traditional ones are based on the ZnEt_2_/H_2_O system used by Inous (Figure 2) [3], while the class of zinc dicarboxylates has shown limited activity [65,66]. A recent approach for heterogeneous catalysts is to develop double metal-based catalysts, such as rare-earth metal ternary complexes and double metal cyanide complexes, which hold good potential for the commercial production of PPC (Figure 4) [67,68,69].

### 3.2. Structure of PPC

The molecular structure of PPC is shown in Figure 2. There are a large number of C-C and C-O single bonds in the main chain, which are easy to rotate and constitute the flexible portion of PPC. In addition, the presence of polar ester motif (-CO-O-) in the backbone not only increases the rigidity of the chain, but also makes the molecule susceptible to hydrolysis. Specifically, the terminal hydroxyl group (-OH) of PPC can attack the ester motif to cause pyrolysis at low temperature, as discussed below.

## 4. Properties of PPC

PPC typically appears as odorless white chunks, but can also be transparent or yellowish. The properties of PPC are mainly determined by its molecular composition, including chain length/molecular weight (Mw), branching and functional groups. Overall, PPC has favorable biodegradability, biocompatibility and barrier properties, but poor aqueous solubility, mechanical properties and thermal stability.

### 4.1. Solubility

PPC is hydrophobic due to the nature of the aliphatic chain. However, it can be dissolved by some organic solvents. The solubilities of PPC film (Mw 4.26 × 10^4^ g/mol; Zhongju, Tianguan, China) in 50 reagents are shown in Table 2. PPC is soluble in two kinds of reagents. The first kind is organic solvents with low to medium polarity (e.g., acetone, dichloromethane, ethyl acetate, aniline, etc.), because the presence of the polar carbonate motif in the backbone confers weak polarity to PPC. The dipole–dipole interaction between PPC and the molecules of low-polarity solvents can facilitate the dissolution of PPC. The second kind is acidic solutions (e.g., 98% hydrochloric acid, 98% sulfuric acid, trifluoroacetic acid). Under such strong acidic conditions, the carbonate groups of PPC are susceptible to nucleophilic attack by hydronium ions (H_3_O^+^), resulting in hydrolysis and formation of CO_2_ and alcohol (mainly 1,2-dipropanol). The poor water solubility of PPC makes it difficult to dissolve in common solutions used in biomedical applications, such as saline and phosphate buffers. In scenarios where an organic solvent is used to dissolve PPC for biomedical applications, the residue organic solvent may inadvertently cause toxicity in the human body. Hence, the hydrophilic modification of PPC to improve its water solubility is desired.

### 4.2. Mechanical Properties

The mechanical properties of PPC are associated with its chemical composition and chain length [5,70]. The presence of carbonate groups contributes to the rigidity and stiffness of the PPC molecule. However, the mechanical properties of PPC vary with the chain length. The reported Young’s modulus of PPC exhibits a wide range of 200–1400 MPa, while the tensile strength is in the range of 7–30 MPa and the elongation at break varies from 600% to 1200% [70]. Moreover, it has been shown that the elongation of PPC chain by ternary or multicomponent polymerization can improve its mechanical properties [4].

Considering the entanglement effect caused by the high aspect ratio of one-dimensional fillers and intermolecular complexation, Wang and co-workers introduced hydroxylated carbon nanotubes (CNTs) and polyvinyl alcohol (PVA) into PPC. This design aimed to restrain the movement of the PPC chain by the hydrogen bonds between PPC, CNTs and PVA. Hence, the introduction of CNTs and PVA resulted in a substantial increase in the mechanical strength of the final ternary composite (PPC/CNT)PVA, reaching 62.7 MPa of tensile strength, more than four times higher than the average tensile strength reported for PPC (15 MPa) [71]. These PPC-based composites with enhanced mechanical properties can be used in implantable materials and wearable electronic devices. In addition, these PPC-based composites can improve the limited mechanical properties of natural polymers, which can further expand the application of PPC as a biomaterial.

### 4.3. Thermal Stability

The thermal property is an important index for PPC [72]. The glass transition temperature (Tg) of PPC is generally low, within the range of 18–40 °C. Meanwhile, PPC is prone to thermal decomposition at temperatures above 150 °C [73,74,75]. As a result, PPC exhibits poor thermal stability and a limited temperature window for fusional processing.

**Figure 5:**
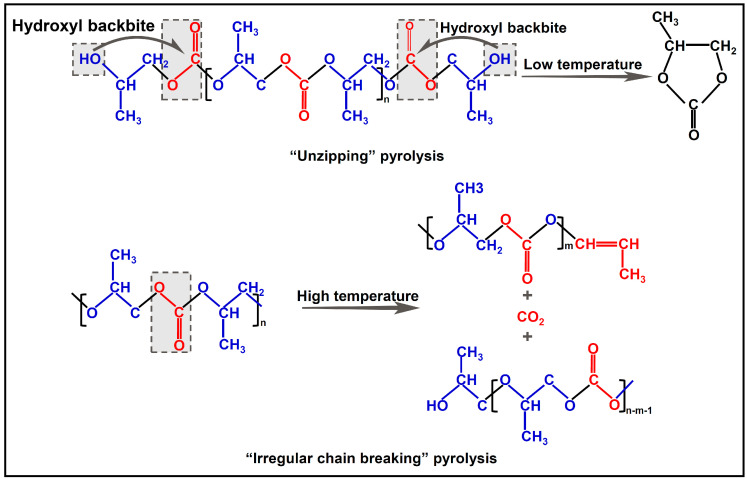
Two types of pyrolysis for PPC: “unzipping” and “irregular chain breaking”. (Blue represents the PO motif of the PPC skeleton. Red represents the ester motifs, CO_2_ or alkene bonds.).

The main reason for its poor thermal stability is because of the low Mw of PPC, which results in relatively weak intermolecular forces. In addition, the presence of a significant number of ester motifs in the backbone, along with the end hydroxyl groups, renders PPC susceptible to hydrolysis and pyrolysis during heating. Overall, there are two types of pyrolytic reactions for PPC, namely, “unzipping” caused by “hydroxyl backbite” and “irregular chain breaking” [4]. Under low temperatures, the “unzipping” degradation can occur while the end hydroxyl group attacks adjacent ester motifs. Such a degradation will be more evident when there is a residual catalyst or the synthesized PPC is not capped at the ends. In contrast, “irregular chain breaking” is dominant at high temperatures, and the final pyrolytic products are CO_2_ and compounds with alkene bonds at the ends (Figure 5).

Currently, there are several methods available to improve the thermal stability of PPC. One approach is to cap the end of PPC or to fix the end hydroxyl groups through ternary or multicomponent polymerization. Maleic anhydride (MA), isocyanates and organosulfur compounds are commonly used capping agents. Grafting PPC with MA has resulted in composites with improved thermal stability and better processability for extrusion at around 150 °C [76]. However, although the end-capping can prevent the “unzipping” of PPC at low temperatures, the “irregular chain breaking” of PPC can still occur at higher temperatures, resulting in the formation of compounds with unsaturated C=C bonds. Moreover, the heat resistance of PPC can also be enhanced by incorporating inorganic or organic compounds. For example, Jiang and co-workers successfully modified PPC with carboxymethylcellulose-boron nitride nanosheets (CMC-BNNS) to enhance the heat resistance and mechanical properties of PPC [75]. In addition to the aforementioned methods, increasing the Mw of PPC and developing catalysts with high conversion rates can reduce the relative content of hydroxyl groups and yield PPC with better thermal properties. One example is the utilization of a double-metal cyanide (DMC) to produce PPC with improved purity and thermal stability through the photopolymerization of PO and CO_2_ [77]. It is worth noting that whatever methods are used, the thermal stability of PPC is directly proportional to its molecular weight. PPC of higher molecular weight typically displays greater thermal stability and thus is more favored for biomedical applications.

### 4.4. Hydrophobicity and Barrier Properties

Overall, PPC is hydrophobic with excellent barrier properties. The water contact angle (WCA) of PPC film is greater than 100° [78], indicating a high level of hydrophobicity. Moreover, PPC exhibits good gas barrier properties. It has been reported that the oxygen permeability and the water vapor permeability of PPC is superior to other biodegradable synthetic polymers, such as PLA [4,79]. For example, Flodberg and colleagues prepared PPC films using a casting method and compared the barrier properties of PPC and PLA films via a special algorithm suitable for the dynamic measurement of oxygen and water vapor transport rates [80]. The oxygen transport rates of PPC film ranged from 10 to 20 cm^3^/m^2^/day/atm, which is significantly lower than that of PLA film (~550 cm^3^/m^2^/day/atm). Similarly, the water vapor transport rate of PPC film was measured as 40–60 g^2^/m^2^/day, also lower than that of PLA film (~300 g^2^/m^2^/day). The barrier properties of PPC can be further improved by incorporating organic or inorganic compounds. For instance, Zhang and co-workers reinforced PPC with laponite and montmorillonite (MMT), and enhanced the oxygen barrier properties of PPC by a factor of 100 compared to pure PPC [81]. Xie and co-workers fabricated nanocomposites of PPC and polytetrahydrofuran-functionalized reduced graphene oxide (PTHF-fRGO), which exhibited improved barrier properties [82]. The barrier properties of PPC against oxygen and water vapor make it a good packaging material for food, pharmaceuticals, medical devices, etc. [5,83,84].

The barrier properties of PPC have a two-fold influence on its biomedical applications. On one hand, PPC exhibits limited integration with cells and tissues under physiological conditions owing to its hydrophobic nature, which hinders cell adhesion and tissue growth. On the other hand, its barrier properties can be advantageous in wound dressings as it creates a conducive hypoxic environment that can benefit cell growth.

### 4.5. Biodegradability

Some studies have shown that PPC is biodegradable without the release of toxic gases under natural conditions [4]. Therefore, PPC is considered as “a green plastic”. However, based on our experience, the biodegradation of PPC mainly occurs at the surface and highly depends on its Mw and the amount of micro-organisms used. Lower Mw will result in faster degradation [85]. For example, the work of Luinstra and co-workers shows that PPC sheets with an Mw of 5 × 10^4^ g/mol can be degraded within 3 months by fermentation with abundant micro-organisms at 60 °C [5]. In contrast, PPC films with a higher Mw of 4.63 × 10^5^ g/mol can only be degraded by 8% after being buried in soil for 6 months [73]. The variation in the biodegradation of PPC is probably also due to the enzymes expressed by different microorganisms. To test this hypothesis, a copolymer film of PPC and PCL, poly(propylene carbonate-co-ε-caprolactone), has been fabricated and placed in phosphate buffer with a panel of enzymes [86]. It has been found that the film can be degraded by Rhizopus arrhizus lipase, ColoneZyme A and Proteinase K.

During the in vitro biodegradation by microorganisms, PPC can be first degraded into oligomers, which can be further decomposed to yield the final products of CO_2_ and H_2_O [4,87]. Hence, one study has shown that the degradation rate of PPC can be determined by CO_2_ production [4]. However, the mechanism of the in vivo biodegradation of PPC remains unclear. Some studies have suggested that PPC undergoes biodegradation primarily through enzymatic hydrolysis while oxidative degradation plays a minor role [88,89]. The enzymatic hydrolysis is considered as an erosive process, whereas oxidative degradation is triggered by the generation of oxidative free radicals from macrophages in response to PPC. Although the degradation mechanism is still under investigation, it has been noted in research that PPC particles partially degraded in abdominal cavities did not cause harm to rats [90]. As aforementioned, the safe degradation products make PPC a good choice of biomaterials.

### 4.6. Biocompatibility

It has been demonstrated that the biodegradation of PPC implanted in rabbits does not elicit adverse tissue necrosis [73,91,92]. Furthermore, PPC has shown superior biocompatibility in certain biomedical applications compared to PLA, which yields acidic products when biodegraded and may cause complications [73]. Hence, PPC has been considered as a promising alternative to PLA-based polymers [73,93,94].

However, the good biocompatibility of PPC only means low cytotoxicity when biodegraded. It does not equate to being integrable by tissues. In fact, the hydrophobic nature of PPC hinders cell and tissue adhesion, thus, limiting its further application as a biomaterial in vivo. Hence, the biomodification of PPC to enhance its interaction with surrounding tissues and promote integration would significantly expand its potential as a biomaterial in vivo. For example, a PPC–starch composite fabricated by melt blending of the PPC and starch has exhibited good cytocompatibility and histocompatibility in mice [93]. After 8 weeks of implantation, PPC–starch only resulted in mild inflammation and was better tolerated compared to PLA.

## 5. Modifications of PPC

**Figure 6:**
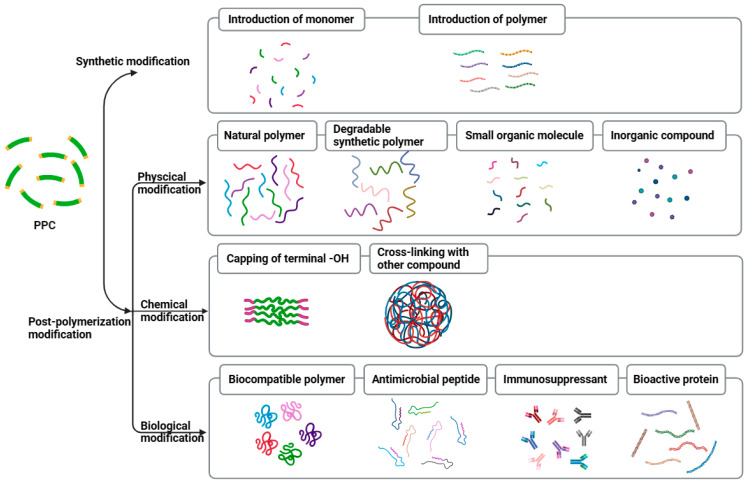
Diagram of PPC modification methods.

Although PPC is considered as a promising polycarbonate derived from CO_2_, its application is still hindered by a low glass transition temperature (less than 50 °C), poor heat resistance and poor cell and tissue adhesion [4,70,73,95]. Hence, various methods have been explored to modify PPC in order to improve its diverse properties to meet the demands of potential applications. These modifications of PPC can be mainly categorized into two approaches, synthetic modification and post-polymerization modification, as depicted in Figure 6 [4,96].

### 5.1. Synthetic Modification

Introduction of a rigid or polar third monomer into the copolymerization of PPC can fundamentally change the main chain structure, thereby enhancing its properties [97]. For example, Meng and co-workers successfully achieved the co-polymerization of PO, CO_2_ and phthalic anhydride under mild conditions. Compared to commercial PPC, PPC with phthalic anhydride (PPC-P) demonstrates superior thermal stability, mechanical properties and degradability [98]. Furthermore, other polymers can also be introduced into the synthesis of PPC to form block copolymers to improve the properties of PPC [96,99]. For instance, Feng and co-workers reported a triblock copolymer of poly(D-lactide)-block-poly(propylene oxide-co-propylene carbonate)-block-poly(D-lactide) (PDLA-*b*-PPPC-*b*-PDLA) utilizing bifunctional ammonium salts as initiators for the copolymerization between CO_2_ and PO and the subsequent ROP of lactide [100]. The triblock copolymer exhibited significantly improved ductility and mechanical properties. The Young’s modulus, tensile strength, elongation at break and toughness of the triblock copolymer exceeded those of commercial high-density polyethylene (HDPE) and low-density polyethylene (LDPE). Although the copolymerization of PO and CO_2_ with a third monomer or polymer can regulate the structure and properties of PPC at the molecular level, it remains a significant challenge to balance the synthesis efficiency, purity and overall performance of synthetically modified PPC.

### 5.2. Post-Polymerization Modification

Post-polymerization modification of PPC roughly includes three methods, i.e., physical, chemical and biological modifications. It should be noted that herein, chemical modification is defined as the modification of PPC molecules in the bulk, whereas biological modification means the surface modification of PPC with bioactive molecules.

#### 5.2.1. Physical Blending

Blending is the major approach for achieving the physical modification of PPC, which is a relatively simple and economical method. Both solution blending and melt blending have been used to prepare PPC-based composites from natural polymers, degradable synthetic polymers, small organic molecules and inorganic compounds [85,101,102,103,104]. For example, Meng et al. obtained a composite of PPC and thermoplastic polyurethane (TPU) by melt blending and discovered that the composites display a transition from brittleness to toughness upon the addition of 20% TPU. Meanwhile, the addition of TPU can significantly improve the heat resistance of PPC [102]. In addition, several inorganic compounds, including calcium carbonate, graphene, montmorillonite, hydroxyapatite and carbon nanotubes, have also been mixed with PPC [11,71,91,103,104]. For instance, Li et al. prepared graphite nanoplates-spherical nanocrystalline cellulose (GNP-SNCC) hybrids from graphite and cellulose through ball milling. Then, GNP-SNCC and PPC were mixed in solution to obtain the composite of PPC/GNP-SNCC. The improved interfacial interaction between GNP-SNCC and PPC, along with the rigid two-dimensional structure of GNP-SNCC, confined PPC molecules, resulting in enhanced thermal stability and mechanical properties [103].

Physical blending is easy to implement in industrial production. However, it does present certain disadvantages. For example, the poor water solubility of PPC hinders its compatibility with water-soluble compounds or polymers. In addition, the high temperature used for melt blending between PPC and other synthetic polymers can lead to degradation and result in reduced mechanical properties and thermal stability compared to individual materials.

#### 5.2.2. Chemical Modification

PPC can be chemically modified by capping and crosslinking reactions to achieve better properties [105]. As aforementioned, the terminal -OH of PPC can trigger the low-temperature pyrolysis of PPC via the “hydroxyl backbite” mechanism. So, capping of the terminal -OH of PPC can prevent the degradation of PPC and is an effective way to increase its thermal stability. For example, Meng et al. used the -COOH of poly(butylene succinate) (PBS) to block the -OH of PPC. Both theoretical calculations and experimental data showed enhanced toughness and strength for the synthesized PPC-mb (multiblock)-PBS [106]. In addition, other capping agents, such as maleic anhydride, benzoyl chloride and isocyanate, have also been utilized to protect the terminal -OH of PPC [107]. Notably, capping of the terminal -OH can improve the mechanical properties and low-temperature stability of PPC, but it does not block the “irregular chain breaking” at high temperatures.

Furthermore, chemical cross-linking can also effectively enhance the thermal stability of PPC [85]. For instance, He et al. prepared a cross-linked PPC network in the form of dioxane gels by cross-linking PPC-OHs with 4,4′-diphenylmethane diisocyanate (MDI) as the cross-linking agent. The cross-linked PPC gels exhibited an enhanced thermal stability [108]. Another important modification of the terminal -OH of PPC is grafting of water-soluble polymers such as PEG [13,109,110], which improves the water-solubility of PPC. Such a hydrophilic modification is critical to PPC-based drug carriers, as discussed below.

#### 5.2.3. Biological Modification

The surface of PPC can also be biologically modified after certain treatments. Surface treatments like plasma, ionizing radiation, laser irradiation or silane coupling can impart hydrophilic surfaces and improve the cell adhesion and biocompatibility for PPC [111,112,113]. For example, the surface of PPC/laponite nanocomposites can form interconnected microporous structures after the treatment with sodium hydroxide, which can boost the surface roughness, surface energy and protein adsorption capacity of PPC [114]. These improvements eventually promote better adhesion, proliferation and differentiation of rat bone marrow mesenchymal stem cells (rBMSCs). Moreover, after surface treatment, PPC can be biofunctionalized with bioactive agents, such as biocompatible polymers, bioactive proteins, antimicrobial peptides and immunosuppressants. For instance, parallel-aligned PPC microfibers, which have been treated with oxygen plasma and surface-modified with chitosan nanofibers, have demonstrated superior cell responses of fibroblasts in terms of morphology, adhesion and proliferation [16]. PPC with plasma treatment and surface modification of hydrogels containing chitosan, collagen and spermidine has exhibited low immunogenicity, which may improve its integration with cell and tissue and favor biomedical applications [78]. However, surface treatment methods may not be suitable for the large-scale production of PPC-based biomaterials.

## 6. Biomedical Applications of PPC

As stated above, PPC holds significant potential for a wide range of medical applications. Indeed, PPC has been used in a variety of medical supplies including masks, surgical gowns, insulating pads and trash bags for medical disposal. In addition to these low-end applications, most of the recent research on PPC-based biomaterials is focused on drug carriers, medical dressings and implants (Table 3), especially for biomodified PPC. In this section, we provide a brief overview of the advances in using PPC as drug carriers, medical dressings, implants and scaffolds.

### 6.1. Drug Carriers

**Figure 7:**
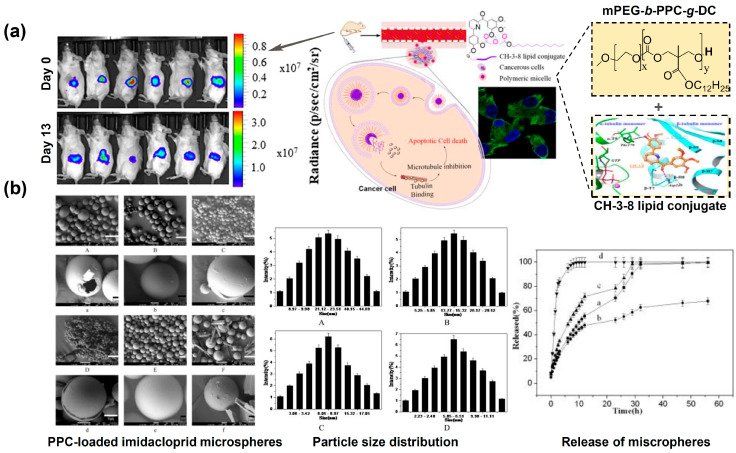
Application of PPC-based biomaterials as drug carriers. (**a**) mPEG-*b*-PPC-*g*-DC polymeric nanoparticles with encapsulated CH-3-8 lipid conjugate (LDC) for cancer therapy [116]; (Left figure represents bioluminescent images at day 1 and day 13 of treatment with LDC in nanoparticles; Right represents the molecular structure of mPEG-b-PPC-*g*-DC, design strategy and drug delivery application of nanoparticles.) (**b**) synthesis and characterization of imidacloprid microspheres for controlled drug release [14]. (Left figure represents SEM image (scale bar: 10 μm) of the microspheres under different shear rate: A, 7000 r/min; B, 10,000 r/min; C, 13,000 r/min; D, 16,000 r/min; E, 10,000 r/min; F, 10,000 r/min; wherein a, b, c, d, e and f drawing of A, B, C, D, E and F partial enlargement, respectively. Middle figure represents particle size distribution of microspheres under the different shear rate: A, 7000 r/min; B, 10,000 r/min; C, 13,000 r/min; D, 16,000 r/min. Right figure represents the release of different particle size microspheres.).

As listed in Table 3, a variety of PPC-based drug delivery systems have been developed, particularly with PEG. Amphiphilic block copolymers composed of PPC and PEG possess favorable thermo-responsiveness and can self-assemble into nanoscale micelles in aqueous solutions, which are promising candidates for drug encapsulation [13,115,116,117,118,119,120,121,122]. One example is shown in Figure 7a. Mahato and co-workers synthesized a methoxy poly(ethylene glycol)-block-poly(2-methyl-2-carboxyl-propylene carbonate-graft-dodecanol) (mPEG-*b*-PCC-*g*-DC) nanoparticle [116]. This nanoparticle can effectively improve the prognosis of pancreatic cancer by overcoming chemotherapy resistance and reducing systemic toxicity. Meanwhile, drug carriers of PPC with other modifications have also been reported, which are normally prepared by electrospinning, melt blending and emulsification and solvent evaporation [14,123,124]. An example is given in Figure 7b. Li and co-workers fabricated PPC-loaded imidacloprid microspheres by emulsion solvent evaporation [14]. The microspheres can achieve a high drug loading of 45%, an entrapment efficiency of 78% and a sustained drug release at shear rate of 10,000 r/min. Furthermore, the targeted delivery of PPC-based drug carriers can also be made by incorporating targeting ligands through biological modification [109,119]. For example, Goutam Mondal and co-workers prepared an epidermal growth factor receptor (EGFR)-targeted gemcitabine (GEM)-conjugated polymeric mixed micelles GE11-PEG-PCD/mPEG-*b*-PCC-*g*-GEM-*g*-DC to treat pancreatic cancer. In mice, GE11-linked micelles can deliver GEM to EGFR-expressing pancreatic cancer cells, act on tumor blood vessels and show significant inhibition of pancreatic tumor growth [119].

### 6.2. Medical Dressings

**Figure 8:**
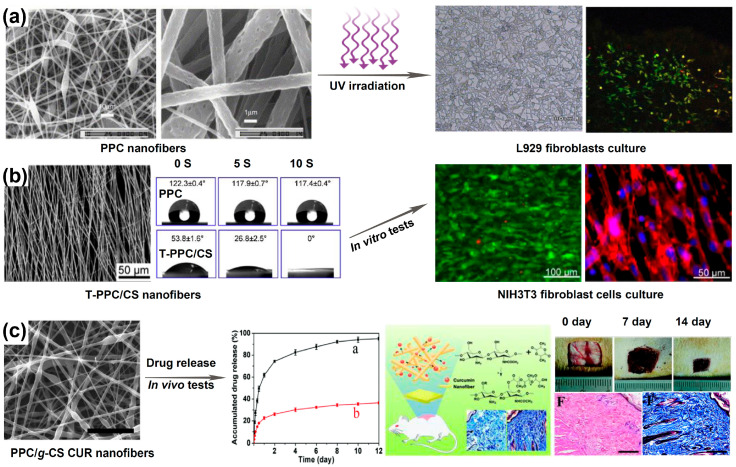
Application of PPC-based biomaterials as wound dressings. (**a**) UV-irradiated electrospun PPC nanofibers with improved cytocompatibility [127]; (**b**) aligned electrospun PPC microfibers treated by plasma and modified with chitosan nanofibers (T-PPC/CS), which are hydrophilic and show enhanced cell attachment, proliferation and cell-scaffold interactions [16]; (**c**) curcumin-loaded PPC/g-chitosan (PPC/*g*-CS CUR) nanofibers as wound dressings [128].

The application of PPC as a wound dressing is unfortunately compromised by its hydrophobicity. Hence, modification of PPC by plasma treatment, UV irradiation and/or polymer grafting is normally used to make PPC-based medical dressings (Figure 8) [16,127,128]. These biomodifications facilitate cell adhesion, proliferation and tissue regeneration while maintaining the essential properties of PPC, such as low toxicity and biodegradability. For example, Alexander Welle et al. prepared PPC nanofibers through electrospinning and subsequent UV irradiation [127]. The UV-irradiated nanofibers exhibited good adhesion and viability of L929 fibroblasts and primary rat hepatocytes, as well as collagen deposition, which show good potential for wound dressings. Peng et al. introduced freeze-dried chitosan nanofibers onto a PPC microfiber mat after oxygen plasma treatment [16]. The composite nanofibers (T-PPC/CS) were hydrophilic and showed superior cell morphology, attachment and proliferation, which makes them suitable for wound dressings. Guo et al. adopted electrospinning to encapsulate curcumin into chitosan-grafted PPC nanofibers [128]. The nanofibers (PPC-*g*-CS CUR) showed granulation and antioxidant effects in animals, which hold great promise for applications in wound repair.

### 6.3. Implants and Scaffolds

**Figure 9:**
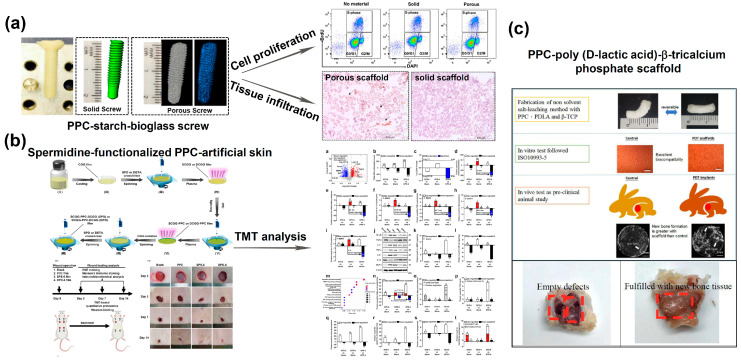
Application of PPC-based biomaterials as implants or scaffolds. (**a**) A biodegradable PPC-starch-bioglass scaffold with improved biocompatibility and tissue integration [15]; (The arrows in H&E stainings represent tissue infiltration.) (**b**) spermidine-functionalized artificial skin to modulate implant-induced immune response and enhance wound healing [78]; (**c**) an osteoconductive PPC-poly(D-lactic acid)-β-tricalcium phosphate (PDT) scaffold for bone defect repair [131].

Among various biodegradable synthetic polymers, PPC is a promising candidate for clinical implants and scaffolds owing to its non-toxic degradation products. Again, various biomodifications have been utilized to prepare PPC-based implants and scaffolds (Figure 9). For example, Fariba Dehghani et al. fabricated a porous scaffold with excellent biocompatibility and benign degradation by-products through gas foaming of PPC blended with starch and bioglass particles [15]. The scaffold demonstrated outstanding cell proliferation and tissue infiltration in vitro and in vivo as well as ideal mechanical properties. Therefore, the scaffold is expected to provide good joint implants. Fang et al. prepared an elastic porous bone scaffold of PPC-poly(D-lactic acid)-β-tricalcium phosphate (PDT) via a non-solvent method [131]. This scaffold not only showed good cytocompatibility and low inflammatory response, but also functioned as an osteogenesis-inducer to promote bone repair in rabbits. Liu et al. modified PPC with biopolymers and spermidine to prepare PPC-based artificial skin [78], which showed excellent mechanical properties, swelling properties, cytocompatibility, and pro-healing properties. More importantly, the PPC-based artificial skin exhibited low immunogenicity owing to the modification of spermidine, which is manifested by reduced pro-inflammatory cytokines in rats and accelerated transition from the M1 macrophage-dominated phase to the M2 macrophage-dominated phase.

### 6.4. Other Biomedical Applications

In addition to the above applications of PPC-based biomaterials, PPC can also be used as a component in the formulation of medical glues for wound closing [132], bio-resistant coatings for antibacterial purposes [92], wearable electronic devices [133] and biomedical instruments [95] to detect various life indicators.

## 7. Other Applications of PPC

In addition to biomedical applications, PPC can also be modified for food packing materials [7,134], UV shielding materials [135], construction materials [136], agricultural mulching films [11], degradable surfactants [137], foaming and blowing materials [138], solid electrolytes and barriers [139,140] and smart materials [104] based on its outstanding properties. For a more comprehensive review of PPC in other applications, please refer to previous reviews [5,85,95].

## 8. Conclusions and Future Perspective

**Figure 10:**
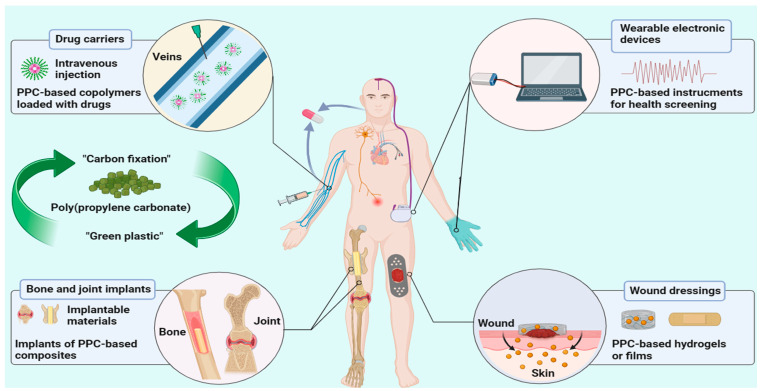
Biomedical applications of PPC-based biomaterials. (The authors thank BioRender for drawing the graphical abstract of the manuscript.).

PPC is known to be an environmentally friendly and biodegradable CO_2_-based polymer that can provide a “carbon fixation” solution to address the challenge of global warming and has the potential to become a renewable resource. Herein, we have reviewed the molecular structure, catalysts for synthesis, properties, modifications and biomedical applications of PPC in detail. In particular, we have highlighted the biomedical applications of PPC-based biomaterials as drug carriers, bone implants, wound dressings and wearable electronic devices (Figure 10).

Although PPC is a promising material for biomedical applications owing to its good biodegradability and safe degradation products, its biomedical application is still constrained by its hydrophobic nature, poor thermal stability and unclear degradation mechanism.

One direction of future research on PPC-based biomaterials is to develop new biomodification strategies. On one hand, such modifications should enhance the hydrophilicity of PPC and facilitate its integration with cells and tissues. On the other hand, the modifications should afford more complicated biological functions. For example, the biomodification of PPC with cell targeting agents may improve the bioavailability of PPC-based biomaterials as drug carriers. The modification with immunoregulatory agents may reduce immune rejection caused by PPC degradation intermediates.

Another important direction is to develop more reliable catalysts for the large-scale production of PPC with a higher molecular weight. As aforementioned, PPC of a higher molecular weight typically displays greater thermal stability and thus is more favored for biomedical applications. Current catalysts for the commercial production of PPC still fails to maintain the long-term stable production of large PPC chains. Hence, novel catalysts for PPC synthesis are still crucial. To this end, double metal-based heterogeneous catalysts are expected to achieve the reliable commercial production of high-molecular-weight PPC.

A third perspective is to investigate the in vivo degradation mechanism of PPC. Although some studies have shown that the degradation products of PPC (CO_2_ and H_2_O) are safe to animals, the specific in vivo degradation mechanism is still unclear and the potential harm caused by the oligomers generated during the degradation also requires investigation. Clinical trials with PPC-based biomaterials should be conducted and organ toxicity should be examined to ensure safe medical applications.

Last but not least, new manufacturing technologies may enable the 3D printing of PPC and thus open the door for the customized application of PPC-based biomedical materials.

## Figures and Tables

**Table 1:** Catalysts for the synthesis of PPC.

	Class	Representative Catalysts	Characteristics	Ref.
Homogeneous catalysts	Zinc complex	(2,6-R_2_C_6_H_3_O)_2_Zn(base)_2_ [R = Ph, ^t^Bu, ^i^Pr, base = Et_2_O, THF or propylene carbonate]; (2,4,6-Me_3_C_6_H_2_O)_2_Zn(pyridine)_2_	(1) Relatively low activity (2) Low polymerization rate (3) Molecular weight (M_W_) of ~45 kg/mol	[53]
		[Zn(OMe)(bdi)]_2_	(1) Turnover frequency (TOF) of Ca. ~350 h^−1^ (2) Low selectivity of PPC (3) Low M_W_ of ~25 kg/mol	[54,55]
	Aluminum porphyrin complex	Al-TPP	(1) Relatively low activity (2) Low polymerization rate and TOF (3) Low M_W_ of ~8900 g/mol	[56]
		Modified Al-TPP	(1) High activity and polymerization rate (2) TOF of Ca. ~560 h^−1^ (3) High selectivity up to 93% (4) High Mw of 96 kg/mol	[57]
	Cobalt^III^ complex	TPP-Co^III^X	(1) TOF of Ca. ~188 h^−1^ (2) High selectivity of PPC (by-product below 1%) (3) Mw of 48–115 kg/mol	[58]
		*N*, *N*′-bis(salicylidene)-1,2 phenylenediamino Co^III^ X	(1) TOF of Ca. ~60 h^−1^ (2) High selectivity of PPC up to 99% (3) Mw of ~40 kg/mol	[59]
		Co(salen)X	(1) High activity (2) High TOF of Ca. ~26,000 h^−1^ (3) High Mw of ~300 kg/mol	[60]
	Oligomers of metal complex	Oligomers of Al-porphyrin	(1) High TOF of Ca. 40,000–50,000 h^−1^ (2) High selectivity of PPC up to 99% (3) Mw of ~200 kg/mol	[62,63]
	Metal-free	Onium halides and alkoxides	(1) TOF of Ca. ~500 h^−1^ (2) Selectivity of PPC up to 97% (3) Mw of 0.5–50 kg/mol	[64]
Heterogeneous catalysts	Zinc-based	Zinc ethyl (ZnEt_2_)	(1) Relatively low activity (2) More by-products and less purity	[3]
		Zinc dicarboxylates (ZnSA, ZnGA, ZnAA, and ZnPA); Zinc glutarates	(1) Relatively low activity (2) TOF of Ca. 20–300 h^−1^ (3) Mw of 12–103 kg/mol	[65,66]
	Double metal-based	Rare-earth metal ternary (ZnEt_2_-C_3_H_8_O_3_-Y(CCl_3_COO)_3_; Y(CF_3_COO)_3_-ZnEt_2_-glycerol; Ln(CCl_3_COO)_3_-glycerin-ZnEt_2_)	(1) More by-products and less purity (2) Mw over 100 kg/mol (3) Carbonate unit content (CU%) of 30–40%	[67,68]
		Zn-Co^III^ double metal cyanide (DMC)	(1) High activity (2) Low CU% (3) Relatively low Mw	[69]

**Table 2:** Solubility of PPC film (2 cm × 2 cm × 0.1 cm) in organic and inorganic reagents.

No.		Reagent	Phenomenon	Solubility
1	Organic reagents	dichloromethane	The film dissolves immediately.	+
2		benzene	The film dissolves immediately.	+
3		trifluoroacetic acid	The film dissolves immediately.	+
4		aniline	The film dissolves quickly with no color change.	+
5		*N*, *N*-dimethylformamide	The film crumbles and dissolves quickly.	+
6		acetone	The film turns white and dissolves from edges.	+
7		triethyl orthoformate	The film turns white and dissolves from edges.	+
8		diethylene benzene	The film turns white and slowly dissolves.	+
9		*N*, *N*-dimethylaniline	The film turns white and slowly dissolves.	+
10		(3-aminopropyl) trimethoxysilane	The film turns white and slowly dissolves.	+
11		ethyl acetate	The film turns white and slowly dissolves.	+
12		methyl methacrylate	The film turns white and slowly dissolves.	+
13		isoamyl acetate	The film turns white and slowly dissolves.	+
14		acetic acid	The film turns white and slowly dissolves.	+
15		propanoic acid	The film turns white and slowly dissolves.	+
16		epichlorohydrin	The film slowly dissolves from edges.	+
17		methyl acet aldehyde	The film crumples and slowly dissolves.	+
18		styrene	The film crumples and dissolves.	+
19		1,4-dioxane	The film crumples and dissolves.	+
20		acrylic acid	The film crumples and dissolves.	+
21		trimethoxy octadecylsilane	The film dissolves slowly.	+
22		*N*, *N*-dimethylacetamide	The film dissolves slowly.	+
23		triethylenetetramine	The film floats on top of the solution.	−
24		glutaric dialdehyde	The film floats on top of the solution.	−
25		dimethicone	The film floats on top of the solution.	−
26		polyethylene glycol	The film floats on top of the solution.	−
27		benzyl alcohol	The film floats on top of the solution.	−
28		hydrazine hydrate	The film floats on top of the solution.	−
29		dimethyl sulfoxide	The film floats on top of the solution.	−
30		formaldehyde	The film sinks in the solution.	−
31		methanol	The film sinks in the solution.	−
32		ethanol	The film sinks in the solution	−
33		n-hexyl alcohol	The film sinks in the solution.	−
34		tert-butanol	The film sinks in the solution.	−
35		2-butoxy ethanol	The film sinks in the solution.	−
36		dibutyl phthalate	The film sinks in the solution.	−
37		tetraethyl orthosilicate	The film sinks in the solution.	−
38		dodecane	The film sinks in the solution.	−
39		Dodecyl triethoxysilane	The film sinks in the solution.	−
40		triethoxypropylsilane	The film sinks in the solution.	−
41		triethoxyoctylsilane	The film sinks in the solution.	−
42		1-bromooctane	The film sinks in the solution.	−
43		(3-aminopropyl) triethoxysilane	The film sinks in the solution.	−
44		n-heptane	The film sinks in the solution.	−
45		lactic acid	The film sinks in the solution.	−
46		2,2,4-trimethylpentane	The film sinks in the solution.	−
47	Inorganic reagents	98% hydrochloric acid	The film’s surface forms bubbles.	+
48		98% sulfuric acid	The film dissolves slowly.	+
49		phosphoric acid	The film sinks in the solution.	−
50		aqueous ammonia	The film floats on top of the solution.	−

+: soluble; −: insoluble.

**Table 3:** Categorical overview of PPC-based materials for biomedical applications.

Suggested Application	Material	Preparation Method	Ref.
Drug carriers for cancer treatment	mPEG-PPC-mPEG/doxorubicin	Grafting copolymerization and drug loading by shear emulsification	[13]
	PEG-PPC-PEG/doxorubicin	Condensation and drug loading by nanoprecipitation	[115]
	mPEG-block-PPC-*g*-dodecanol/CH-3-8 polymeric nanoparticles	Grafting copolymerization and drug loading by coupling reaction	[116]
	mPEG-block-PPC-*g*-gemcitabine-*g*-dodecanol/miR-205 polymeric micelles	Grafting copolymerization and drug loading by coupling reaction	[117]
	PEG-block-PPC-*g*-tetraethylenepentamine/GDC-0449/let-7b micelles	Grafting copolymerization and drug loading by coupling reaction	[118]
	GE_11_ peptide-PEG-block-PPC-*g*-gemcitabine-*g*-dodecanol mixed micelles	Grafting copolymerization and drug loading by coupling reaction	[119]
Drug carriers for hepatic fibrosis treatment	mPEG-block-PPC-*g*-dodecanol-*g*-tetraethylenepentamine/miR-29b1/GDC-0449 micelles	Grafting copolymerization and drug loading by coupling reaction	[120]
	mPEG-block-PPC-*g*-dodecanol-*g*/MDB5 micelles	Grafting copolymerization and drug loading by coupling reaction	[121]
Drug carriers for type I diabetes treatment	mPEG-block-PPC-*g*-dodecanol-*g*-tetraethylenepentamine/sunitinib micelles	Grafting copolymerization and drug loading by coupling reaction	[122]
Drug carriers for spinal cord injury treatment	PPC/dibutyryl cyclic adenosine monophosphate/chondroitinase ABC microfibers	Electrospinning	[123]
Drug carriers for other treatments	PPC/PCL/metoprolol tartrate blends	Melt blending	[124]
	PPC/imidacloprid microspheres	Emulsification and solvent evaporation	[14]
	Poly(vinyl-cyclohexene carbonate)-*g*-PPC	Grafting copolymerization	[125]
	PPC-block-poly(4-vinylcatechol acetonide) copolymers	Grafting copolymerization	[126]
Wound dressings	Parallel-aligned PPC microfibers/chitosan nanofibers	Electrospinning and oxygen plasma treatment	[16]
	PPC nanofiber mats	Electrospinning, spin coating and UV treatment	[127]
	Curcumin-loaded PPC-*g*-chitosan nanofibers	Electrospinning and encapsulation	[128]
Artificial skins	Spermidine-functionalized PPC composite films	Spin coating	[78]
Bone repair scaffolds	Porous PPC-starch-bioglass scaffolds	Gas foaming	[15]
	PPC-starch composites	Melt blending	[93]
	Microporous PPC/laponite nanocomposites	Melt blending and surface treatment with sodium hydroxide	[114]
	PPC-starch-bioglass blends	Melt blending	[129]
	PPC multilayer membranes	Aminolysis and layer-by-layer assembly	[130]
	Porous PPC/poly(D-lactic acid)/β-tricalcium phosphate scaffolds	Salt leaching	[131]
Medical adhesives/glues	Poly(ethyl cyanoacrylate)/PPC/caffeic acid films	Polymerization in presence of PPC and solvent evaporation	[132]
Wearable electronic devices	Poly(methyl methacrylate)-PC-lithium perchlorate/multi-walled carbon nanotube/Mn_3_O_4_ micro-supercapacitors layer-by-layer-assembled films	Hydrothermal reaction, photolithography and layer-by-layer assembly	[133]

## Data Availability

Data sharing not applicable.
